# “Because This Is an Evidence-Based Program”: The Learning Experience of Croatia with the Lions Quest Skills for Adolescence, Implemented In-Person and Online

**DOI:** 10.3390/ijerph192214938

**Published:** 2022-11-13

**Authors:** Ziad El-Khatib, Alma Rovis Brandic, Ali Yassine, Zana Glavendekic, Wadih Maalouf

**Affiliations:** 1 Department of Global Public Health, Karolinska Institutet, 171 76 Stockholm, Sweden; 2 World Health Programme, Université du Québec en Abitibi-Témiscamingue (UQAT), Rouyn-Noranda, QC J9X 5E4, Canada; 3 High School Department, Agency for Education, 10000 Zagreb, Croatia; 4 Drug Prevention and Health Branch, Prevention Treatment and Rehabilitation Section, United Nations Office on Drugs and Crime, Vienna International Center, P.O. Box 500, 1400 Vienna, Austria; 5 Programme Office of Serbia, United Nations Office on Drugs and Crime (UNODC), Bulevar Zorana Djindjica 64, 11070 Belgrade, Serbia

**Keywords:** prevention, Croatia, Lions Quest Skills, empowerment

## Abstract

*Background*: The Lions Quest Skills for Adolescence (LQSFA) is an evidence-based social and emotional learning program for school students. It is implemented as a teacher-led extracurricular activity for children aged 10–15 years. From 2019 to 2022, the United Nations Office on Drugs and Crime, in collaboration with Lions Clubs International Foundation, implemented the LQSFA in 41 schools in Croatia. Due to the COVID-19 lockdown measures, the intervention was adjusted into a hybrid modality (in-class and online). We evaluated the experience that the teachers had with the LQSFA in a hybrid modality. *Methods*: We used a focus-group discussion approach to evaluate the experience of five LQSFA teachers. *Results*: Three themes emerged: (1) the appreciation of evidence-based programs by the teachers, (2) the benefit of the LQSFA on the parents, and (3) the length of the online version of the questionnaire tool that was used to assess pre- and post-LQSFA experiences among students was too long. These results indicate that the LQSFA is undergoing a scaling on a national level in Croatia, even when implemented in a hybrid setting. *Conclusions*: Using an evidence-based program such as the LQSFA was rewarding for teachers, despite the challenges in the administrative adjustments regarding the online and in-person class teaching. LQSFA filled an important gap during COVID19-related stress.

## 1. Introduction

The Lions Quest Skills for Adolescence (LQSFA) is an evidence-based social and emotional learning program for school students [1,2,3]. It was developed by the Lions Clubs International Foundation and targets the 10–15 age group. In 2010, the United Nations Office on Drugs and Crime (UNODC) implemented the first LQSFA program in five Southeastern European countries: Bosnia and Herzegovina, Croatia, Montenegro, North Macedonia, and Serbia. The program included a total of 40 sessions over two academic years, in relation to seven topics: (i) entering the teen years, (ii) building self-confidence and communication skills, (iii) managing emotions in positive ways, (iv) improving peer relationships, (v) strengthening family relationships, (vi) living healthy and drug free, and (vii) setting goals for healthy living.

The effect that LQSFA had on recent substance use (alcohol, tobacco, and marijuana) and the intention for future use over the course of one academic year was first evaluated in Serbia, Montenegro, and North Macedonia [2]. Plans were then set to replicate this model in other neighboring countries, including in Croatia, with the intention of implementing all 400 sessions of the LQFSA over two academic years. In 2019, the LQSFA was launched in 41 schools in Croatia, with a plan to be implemented over the course of two academic years. In March 2020, a few months after the beginning of the first academic year, schools started implementing distance learning due to the Coronavirus (COVID-19) pandemic. This had an impact on the LQSFA implementation modality, which was originally designed for class-based environments. The program sessions were designed and structured to develop relevant social skills, including interactions and direct contact between the students. This in-person modality is essential for the students to build these skills, and it is aligned with the guidelines from the UNODC-World Health Organization (UNODC-WHO) International Standards on Drug Use Prevention [4]. Furthermore, the COVID-19 pandemic had a negative impact on the prioritization of the LQSFA program. This came as a result of the difficulties and challenges schools faced in accommodating shifts in the educational system, especially the shift from in-person to online learning. However, in Croatia, the teachers were still interested in conducting the LQSFA, despite the COVID-19 related challenges [1]. Thus, they proceeded with a hybrid implementation of the program. In July 2022, at the end of the second academic year of the LQSFA, the UNODC collaborated with the Education and Teacher Training Agency of the Government of Croatia (TAGC) to evaluate the experience that teachers had with the LQSFA before, during, and after the COVID-19-related lockdowns, including their experience with a questionnaire administered to the students that was used to evaluate the program at pre- and post-intervention.

## 2. Materials and Methods

In July 2022, we planned a mini-focus group discussion (FGD) with 10 social pedagogues. They worked as counselors in their respective schools. They were the designated coordinators from the TAGC. These teachers helped UNODC and the TAGC in coordination of the implementation in the 41 schools in Croatia (among a total of 75 teachers included in the LQSFA). All 75 teachers took part in a total of four follow-up sessions facilitated by UNODC and the TAGC to exchange their experiences (one day per session per region).

On the day of the FGD, 3/10 teachers were infected with COVID-19 and 2/10 were exposed to COVID-19 patients. Therefore, the final FGD was conducted with 5/10 teachers for a duration of 75 min. All teachers were social pedagogues and certified national trainers for the LQSFA program. The meeting aimed to gain a deeper understanding of their experience with the LQSFA in their respective schools. During the FGD, the moderator had open questions about the experiences of the teachers with LQSFA. The FGD approach was used to collect user views and experiences to inform the scaling of the LQSFA process in Croatia. This qualitative method was considered the ideal way to collect instrumental documentation of the experiences, shared thoughts, feelings, and attitudes from all teachers’ practices, and it also facilitated a problem-solving approach for future planning of the LQSFA [5]. The FGD was planned and approved by the Ministry of Education and the TAGC.

## 3. Results

The five teachers involved in the FGD shared their personal experiences, as well as the experiences obtained from their peers during the previous four follow-up sessions. The teachers were structured and organized in their thoughts as they had participated in similar sessions among their peers during the LQSFA process. Therefore, the moderator was flexible and open in the approach of the FGD to give the teachers the freedom to share their views and experiences with LQSFA. The data generated from the FGD were categorized into three themes.

**Theme 1: The appreciation of the need for ‘evidence-based’ programs by the teachers**

Participants shared that the LQSFA was the best quality evidence-based substance use prevention program used in Croatia, especially under such unprecedented circumstances generated by the COVID-19 pandemic. The other schools, enrolled under the control study group (i.e., they were not using the LQSFA), provided self-designed sporadic and non-systematic life skills sessions to their students. These sessions were done in an ad hoc fashion and were not based on evidence of best practice (“*The schools, in the control study group, use life skills sessions. It is not evidence based*”; “*The life skills sessions are mainly in the form of lectures, but not done systematically, or in an interactive manner with the students*”). The subject teachers in Croatia are expected to support the students during their emotional transitions during this critical age of development, although they lack the expertise or the correct skills to tackle it (“*Subject teachers in Croatia are trained on pedagogical skills, and they are experts of their topics. However, they are not equipped with the tools to teach social and emotional learning skills. LQSFA provides these skills to the teachers so they can train the students.*”). All the participants shared a mutual consensus about the value added by LQSFA, as follows: (i) LQSFA empowers students and improves the student–student and student–teacher relationships (“*Students liked the activities. When trainers come to the class, students know they will learn skills and feel excited and happy about it.*”); (ii) LQSFA is a unique and rare substance use prevention package in Croatia and is an evidence-based program, particularly as it was piloted to better fit the Croatian context and tested and evaluated in the context of Croatia (“*I like LQSFA because it is an evidence-based program. I know the material is developed based on evidence*.”).

**Theme 2: The benefit of the LQSFA for the parents**

Parents were briefed about the LQSFA prior to its launch (and prior to the COVID-19 pandemic and school lockdowns) during the yearly parent-teacher meeting in 2019. During the COVID-19 pandemic, the schools could not hold their yearly parent-teacher meeting; however, the teachers received phone calls from the parents regarding the LQSFA, where parents volunteered to share their feedback on the package. While not anticipated in the original modality, one of the worthwhile spinoffs of the lockdown-based implementation of LQSFA was the exposure of parents to the program, which happened for two reasons: (i) during the lockdowns, parents and students shared the same space during the online classes and they could listen, in a passive fashion, to the content of the LQSFA; (ii) the students initiated the discussions with their parents regarding the content of the LQSFA, and the parents reported that they felt content with the content of the LQSFA material. (“*The parents were exposed in a passive way to LQSFA, in two ways: (1) during lockdown, parents could listen to LQSFA sessions in the background, when students were attending it online, and (2) children talked to their parents about the LQSFA sessions. The parents called schools to give feedback. Parents appreciated the skills that were taught to the children, especially the topics of stress management, and coping strategies where they found it useful specially during COVID-19*”).

**Theme 3: Feedback on the LQSFA questionnaire**

The teachers reported that the questionnaire was long for their students. Currently, the questionnaire includes a total number of 67 questions. Prior to the launching of the intervention, the duration of filling out the questionnaire ranged between 45 and 60 min in the classroom. Due to COVID-19 related lockdowns, the questionnaire for the post-LQSFA program was provided online for the students. The online questionnaire provided feedback that the average duration of answering the questionnaire ranged between 7 and 10 min (“*We can monitor the duration spent per questionnaire, in an anonymous way, which means we can’t identify the name of the person who filled in the questionnaire. The students spent a short period of time to fill in the online questionnaire, therefore I suspect the students didn’t read the questions well. The students may have felt bored during the process of filling out the questionnaire*”). On the first page of the questionnaire, a code number was generated for each student, which anonymised the data. The teachers shared that the students faced challenges in their ability to answer certain questions (“*Some students were not sure about the colour of their eyes; Some students used nicknames for parents and some did not know the names of their fathers*”). The teachers also shared that many students did not know what cocaine was, and they became curious to learn about this topic after reading about it in the questionnaire. The teachers suggested to consider deleting this question from the questionnaire (“*Students had not heard about crack, cocaine and heroine before. They became curious about this topic. They either asked their parents, or they searched online. It is an early stage for them to use cocaine or heroine. We suggest to take out the questions related to this substance.*”). Moreover, the teachers received questions from the students asking to clarify the approach of assessing the substance use history of the students (“*Sometimes, the formulation of the questions did not read in a clear way. For example, in a question where it asks about the intention to drink alcohol or to smoke cigarettes and marijuana during the next 3 months; The students asked us what if they had a sip of beer, then would this act count as a drink? Or another question about how to count the act of drinking one time if it was a one sip of champagne on the new year or a sip of beer?*”*).* Finally, the teachers suggested to reduce the number of questions so that it does not exceed a total of 25 questions.

## 4. Discussion

Globally, the COVID-19 pandemic has had a major impact on students, teachers, and parents, as well as different school health promotion activities. Therefore, the skills provided in the LQSFA are helpful during such a period due to the increased burden to mental health on students, teachers, and parents. At the beginning of the pandemic, we expected the LQSFA to face difficult obstacles due to the different administrative measures and policies (e.g., lockdowns and online school classes) [1]. The purpose of this study was to synthesize the perception of the LQSFA delivered in a hybrid fashion by the school teachers in Croatia. This mixed mode of information delivery (online and in-person) was done for the first time due to the forced circumstances related to the COVID-19 lockdown measures. Our findings echo the findings from our previous paper [1] and indicate that all teachers (i.e., not only those exposed to LQSFA) share the interest of having life skills and knowledge passed on, especially during the pandemic. However, other classes taught outside the scope of LQSFA were not evidence-based, and may therefore be less impactful. Moreover, it was interesting to observe the additional positive impact on the parents. This reflects the parents’ increased affinity to and acceptance of the LQSFA program (and, potentially, the parents’ support for the application of these skills at home).

The unique element of this study was that all social pedagogues had a university diploma in pedagogy and could give deeper insights into the value that LQSFA provided. This included the evidence-based material. The UNODC supports several prevention packages that are evidence-based, which are implemented in different countries [4]. It is important to note that the participants were certified national trainers and they were rolling out the LQSFA across the rest of Croatia [6]. However, we should mention one limitation of the study. The data were collected after a challenging three years of COVID-19. Therefore, it was not possible to have quantitative data to assess the impact of the LQSFA on substance use reduction.

With regard to the questionnaire, it was initially designed as a class-based survey but we had to transfer it to an online survey, without the chance to pilot it, due to the fast shift from class-based to online teaching at the beginning of the COVID-19 pandemic [1]. We need to address this concern in the future in case of future COVID-19-related lockdowns. A panel of expert researchers has suggested the maximum length of an online survey should range between 15 and 30 min [7], and we will therefore take this into consideration, in addition to the adaptation to the context of the country. Moreover, we should mention that we did not assess the impact of LQSFA on the transition of children back into class-based learning. However, the LQSFA managed to establish a mutual relationship between the teachers, the parents, and the students, which is one of the main international recommendations for a strong transition post-COVID-19 [8,9,10]. Finally, this report is novel in terms of sharing the experience of LQSFA from the perspective of the teachers in Croatia, and it indicates that this program is scaling on a national level. However, we would like to point out that this study only included one FGD, which was held for a relatively short period of time in Croatia’s relatively good infrastructure context. Due to the COVID-19 circumstances, we could not have the meeting with a larger number of teachers or for a longer period of time. Additionally, due to the nature of the qualitative methods, it is not possible to generalize the findings of this report.

## 5. Conclusions

Evidence-based education material such as LQSFA was considered a successful approach, even when provided in a hybrid program due to the COVID-19 lockdowns.

## Data Availability

Data is available per the communication with the authors.
